# Homocysteine and all-cause mortality in hypertensive adults without pre-existing cardiovascular conditions: Effect modification by *MTHFR* C677T polymorphism

**DOI:** 10.1097/MD.0000000000005862

**Published:** 2017-02-24

**Authors:** Benjamin Xu, Xiangyi Kong, Richard Xu, Yun Song, Lishun Liu, Ziyi Zhou, Rui Gu, Xiuli Shi, Min Zhao, Xiao Huang, Mingli He, Jia Fu, Yefeng Cai, Ping Li, Xiaoshu Cheng, Changyan Wu, Fang Chen, Yan Zhang, Genfu Tang, Xianhui Qin, Binyan Wang, Hao Xue, Yundai Chen, Ye Tian, Ningling Sun, Yimin Cui, Fan Fan Hou, Jianping Li, Yong Huo

**Affiliations:** a Department of Cardiology, Peking University First Hospital, Beijing; b National Clinical Research Study Center for Kidney Disease; State Key Laboratory for Organ Failure Research; Renal Division, Nanfang Hospital, Southern Medical University, Guangzhou; c Institute for Biomedicine, Anhui Medical University; d Department of Neurology, First Affiliated Hospital of Anhui Medical University, Hefei; e Department of Cardiology, the Second Affiliated Hospital of Nanchang University, Jiangxi; f Department of Neurology, First People's Hospital, Lianyungang; g Department of Neurology, Guangdong Provincial Hospital of Chinese Medicine, Guangzhou; h Department of Cardiology, Beijing Anzhen Hospital, Capital Medical University, Beijing; i School of Health Administration, Anhui Medical University, Hefei; j Department of Cardiology, Chinese People's Liberation Army General Hospital, Beijing; k Department of Cardiology, The First Affiliated Hospital of Harbin Medical University, Harbin; l Department of Cardiology, Peking University People's Hospital; m Department of Pharmacy, Peking University First Hospital, Beijing, China.

**Keywords:** all-cause mortality, Chinese, homocysteine, hypertension, *MTHFR* polymorphism

## Abstract

**Background:**

Previous studies support an association between elevated total homocysteine (tHcy) levels and increased all-cause mortality. However, few prospective studies have examined this association in hypertensive patients, and/or tested any effect modification by the methylene tetrahydrofolate reductase (MTHFR) C677T genotype.

**Methods:**

This was a post hoc analysis of the China Stroke Primary Prevention Trial. Serum tHcy and folate were measured at baseline. Individual *MTHFR* C677T genotype (CC, CT, and TT) was determined. Evidence for death included death certificates or home visits. Cumulative hazards of all-cause mortality by tHcy quartiles were estimated using the Kaplan–Meier method, and group differences were compared by log-rank tests. Hazard ratios (HRs) and 95% confidence intervals were estimated by Cox proportional-hazard regression models, adjusting for age, sex, baseline folate, vitamin B12, blood pressure, body mass index, smoking and alcohol drinking status, study center, total cholesterol, triglycerides, high-density lipoprotein cholesterol, fasting glucose, creatinine, and treatment group. Potential effect modification by the *MTHFR* genotype on the relationship between tHcy and all-cause mortality was tested.

**Results:**

The analyses included 20,424 hypertensive patients (41% males) without a history of myocardial infarction or stroke. Baseline mean age (SD) was 60 ± 7.5 years and mean (SD) serum tHcy was 14.5 ± 8.4 μmol/L. After a mean follow-up period of 4.5 years, there were 612 (3%) all-cause deaths. Kaplan–Meier survival curves revealed a graded relationship between tHcy quartiles and all-cause mortality. The HRs, using the lowest quartile as the reference, were 1.2, 1.2, and 1.5 in Q2, Q3, and Q4, respectively. A linear trend test, using natural log-transformed tHcy, resulted in an HR of 1.5 (95% confidence interval 1.2–1.9, *P* < .001) after adjustment for lifestyle and health-related variables. Whereas the *MTHFR* genotype alone had little effect on mortality, it significantly modified the tHcy–mortality association, which was much stronger in the CC/CT genotype than in the TT genotype (*P* for interaction < 0.05).

**Conclusions:**

Among Chinese hypertensive patients without cardiovascular comorbidities, elevated tHcy was a significant risk marker for death from all causes, and the association was subject to effect modification by *MTHFR* genotypes. If confirmed that tHcy and *MTHFR* genotypes may serve as useful biomarkers for mortality risk assessment and targeted intervention.

## Abstract 2

Supplemental Digital Content is available in the text

## 1 Introduction

Elevated levels of blood homocysteine (Hcy) is a well-recognized and modifiable risk factor for cardiovascular disease.^[1]^ As early as 1969, McCully^[2]^ linked severe hyperhomocysteinemia with atherosclerosis and vascular thrombosis. Subsequent research has demonstrated an association between mildly to moderately elevated Hcy, and coronary, cerebral, and peripheral vascular diseases.^[1,3–5]^

Compared with cardiovascular events, all-cause mortality, despite its public health importance, has been less well-studied in homocysteine research. In the past 20 years, research, mostly in western populations, has indicated a link between elevated Hcy and increased mortality.^[6–8]^ For example, plasma total homocysteine (tHcy) levels conferred a graded and independent risk of all-cause mortality among 587 patients (median age 62 years) with coronary artery disease in Norway.^[6]^ Elevated plasma tHcy (14.26 or higher μmol/L) increased all-cause mortality by 50% among 1933 older (mean age 70 years) Framingham men and women.^[9]^ Elevated plasma tHcy (14 or higher μmol/L) increased total mortality in a community based study in Israel.^[10]^ However, there are still important knowledge gaps in this field.

First of all, there is lack of data on whether elevated tHcy is an independent predictor of all-cause mortality in hypertensive patients. Previous studies have shown that tHcy and hypertension can synergistically increase the risk of cardiovascular disease and stroke.^[11]^ Patients with “H-type hypertension,” a term to describe hypertensive patients with elevated homocysteine, are a subgroup at a particularly high risk of stroke.^[12]^ In a subgroup analysis (n = 166) of an earlier study, elevated tHcy was associated with all-cause mortality in the hypertensive participants, but not in the normotensive participants.^[8]^ Clearly, more study is needed to examine this possibility.

Notably, previous studies found that the relationship between tHcy and mortality varied by the participant's pre-existing medical conditions. For example, in a Netherlands study, the Hcy–mortality association was significantly greater in type 2 diabetic patients than in nondiabetic patients (odds ratio [OR] 2.5 vs 1.3).^[13]^ No association was found between tHcy and mortality in patients with chronic kidney disease in a US population.^[14]^ As such, to answer the aforementioned question, an ideal study design would be to assess tHcy–mortality association in hypertensive patients without other cardiovascular comorbidities. Such a study is yet to be conducted.

Secondly, methylene tetrahydrofolate reductase (MTHFR) is a critical enzyme involved in the Hcy metabolic cycle.^[15]^ Mutation from the wild type of *MTHFR* at position 677 from C to T impacts the activity of the MTHFR enzyme. The TT mutation causes the encoding of a thermolabile enzyme to reduce its activity by 70%. Whereas studies have examined the impact of the *MTHFR* genotype on tHcy levels,^[8,16]^ few studies have examined the effect modification of *MTHFR* gene polymorphism on Hcy–mortality association, in part, due to a lack of large-scale cohort studies with data on individual tHcy, *MTHFR* genotype, and mortality.

Finally, few tHcy–mortality studies have been conducted in Asian populations including Chinese populations. There are over 300 million hypertensive patients in China alone. Compared with western populations, Chinese populations have a much higher prevalence of elevated tHcy. In particular, about 75% of hypertensive patients have elevated tHcy.^[17]^ In addition, the *MTHFR* 677TT gene variant is present in about 25% of the Chinese population, again, much higher than that of western populations. These characteristics have made China a compelling setting to examine the independent and joint effect of tHcy and *MTHFR* C677T polymorphism on all-cause mortality. Findings from such a study have important clinical and public health implications, for example, identification of high-risk individuals and development of more effective personalized prevention.

Using data from the China Stroke Primary Prevention Trial (CSPPT, N = 20,424),^[17]^ this study aimed to examine the longitudinal association between tHcy and 5-year all-cause mortality in Chinese adults with hypertension, but without history of stroke or myocardial infarction (MI). It further tested the hypothesis that the association between tHcy and all-cause mortality can be modified by the *MTHFR* C677T polymorphism.

## 2 Methods

### 2.1 Study participants and design

This study included 20,424 participants of the CSPPT (clinicaltrials.gov identifier: NCT00794885) with complete data on baseline tHcy and *MTHFR* genotype. All participants had hypertension, but with no pre-existing cardiovascular or cerebral vascular conditions. The CSPPT was approved by the ethics committee of the Institute of Biomedicine, Anhui Medical University, Hefei, China (FWA assurance number FWA00001263). Written informed consent was obtained from all participants.

Details regarding the study design, study protocol, and field data collection of the CSPPT have been published previously.^[17]^ Briefly, the CSPPT was a randomized, double-blind, controlled trial conducted from May 2008 to August 2013 in 32 communities in Jiangsu and Anhui provinces, China. It aimed to evaluate whether a combined therapy of enalapril plus folic acid (FA) is more effective than enalapril alone in reducing the risk of stroke in hypertensive patients. The CSPPT enrolled men and women aged 45 to 75 years who had hypertension, defined as seated resting systolic blood pressure (SBP) ≥140 mm Hg or diastolic blood pressure (DBP) ≥90 mm Hg at both the screening and the recruitment visit, or who were on antihypertensive medications. The major exclusion criteria included history of physician-diagnosed stroke, MI, heart failure, postcoronary revascularization, or congenital heart disease. Eligible participants, stratified by *MTHFR* C677T genotypes (CC, CT, or TT), were randomly assigned to 1 of 2 treatment groups: a daily oral dose of 1 tablet containing 10 mg enalapril and 0.8 mg FA; or a daily oral dose of 1 tablet containing 10 mg enalapril only. During the trial period, concomitant use of other antihypertensive drugs (mainly calcium channel blockers or diuretics) was allowed, but B-vitamin supplementation was not. Participants were scheduled for follow-up every 3 months until completion of the trial. Each visit included measuring and recording of blood pressure (BP), pulse, treatment compliance, concomitant use of other medications, adverse events, and study outcome events.

### 2.2 Blood pressure measurements

These details have been previously published.^[18]^ Briefly, SBP/DBP (mm Hg) was measured at baseline and at each follow-up visit with the participant in a seated position after having rested for more than 5 minutes, and using a mercury sphygmomanometer with an appropriate cuff size. At each visit, 3 consecutive measurements were obtained on the right arm, with 1-minute intervals between each, and the mean value was calculated. The mean SBP and DBP over the treatment periods were also calculated.

### 2.3 All-cause mortality

All-cause mortality, a prespecified endpoint of the CSPPT, was the main outcome in this analysis and defined in previous publications.^[17,19]^ All-cause mortality includes death due to any reason. Evidence for death included death certificates from hospitals or reports after a home visit by a trained investigator. All-cause deaths were reviewed and adjudicated by an independent Endpoint Adjudication Committee, whose members were unaware of treatment-group assignments.

### 2.4 Laboratory tests

As described in previous publications, overnight fasting venous blood samples were collected at baseline.^[17,20]^ Laboratory tests were performed at the core laboratory of the National Clinical Research Center for Kidney Disease (Nanfang Hospital, Guangzhou, China). Fasting plasma glucose, fasting lipids (serum total cholesterol, high-density lipoprotein cholesterol [HDL-C], and triglycerides), serum tHcy, and creatinine were measured using automatic clinical analyzers (Beckman Coulter). Serum folate and vitamin B12 were measured at baseline by a commercial laboratory using a chemiluminescent immunoassay (New Industrial). *MTHFR* C677T (rs1801133) polymorphisms were detected on an ABI PRISM 7900HT sequence detection system (Life Technologies, CA) using the TaqMan assay.

### 2.5 Covariates

Covariates included age, sex, body mass index (BMI) (weight kg/height m^2^), baseline SBP and DBP, mean SBP and DBP during treatment period, baseline serum total cholesterol, HDL-C, triglycerides, fasting glucose, creatinine, folate, vitamin B12, smoking status, alcohol consumption, and treatment group.

### 2.6 Statistical analysis

Means (standard deviation [SD]) or proportions were calculated for population characteristics by tHcy quartiles, the major exposure variable. All-cause mortality over the course of the follow-up period was the main outcome of interest. Cumulative hazards of all-cause mortality by tHcy quartiles were estimated using the Kaplan–Meier method, and group differences were compared by log-rank tests. Hazard ratios (HRs) and 95% confidence intervals (CIs) were estimated by Cox proportional-hazard regression models, adjusting for important covariables, including age (<55, 55 to <65, and ≥65), sex, baseline folate quartiles, vitamin B12 quartiles, SBP at baseline (<160, ≥160 mm Hg), BMI (<25, ≥25 kg/m^2^), smoking and alcohol drinking status, study center, total cholesterol, triglycerides, HDL-C, fasting glucose, creatinine, and treatment group. Potential effect modification by the *MTHFR* genotype on the relationship between tHcy and all-cause mortality was evaluated by stratified analyses and by interaction testing. A 2-tailed *P* < 0.05 was considered to be statistically significant in all analyses. R software, version 3.2.0 (http://www.R-project.org/) and Empower (R) (www.empowerstats.com, X&Y Solutions, Inc. Boston, MA) were used for all statistical analyses.

## 3 Results

### 3.1 Study participants and baseline characteristics

The CSPPT had a total of 20,702 participants. After excluding 278 participants without baseline tHcy, this study included 20,424 participants for the final analysis. Table 1 presents demographic, clinical, and laboratory characteristics of the study participants by baseline tHcy quartiles. The average age was 60.0 years and 41% were male. All participants were hypertensive patients, with a mean baseline SBP of 167 and DBP of 94 mm Hg. None of the participants had a prior history of MI or stroke. Over the course of treatment, mean SBP was 139 and mean DBP was 83 mm Hg. The frequency of the *MTHFR* C677T polymorphism was 27% for CC, 49% for CT, and 24% for TT, a genotype distribution that is consistent with the Hardy–Weinberg equilibrium. The mean (±SD) baseline tHcy levels were 14.5 ± 8.6 μmol/L in the enalapril group and 14.4 ± 8.1 μmol/L in the enalapril + FA group. Higher levels of tHcy were associated with older age, male sex, elevated serum creatinine levels, lower folate levels, and the *MTHFR* TT genotype.

**Table 1:**
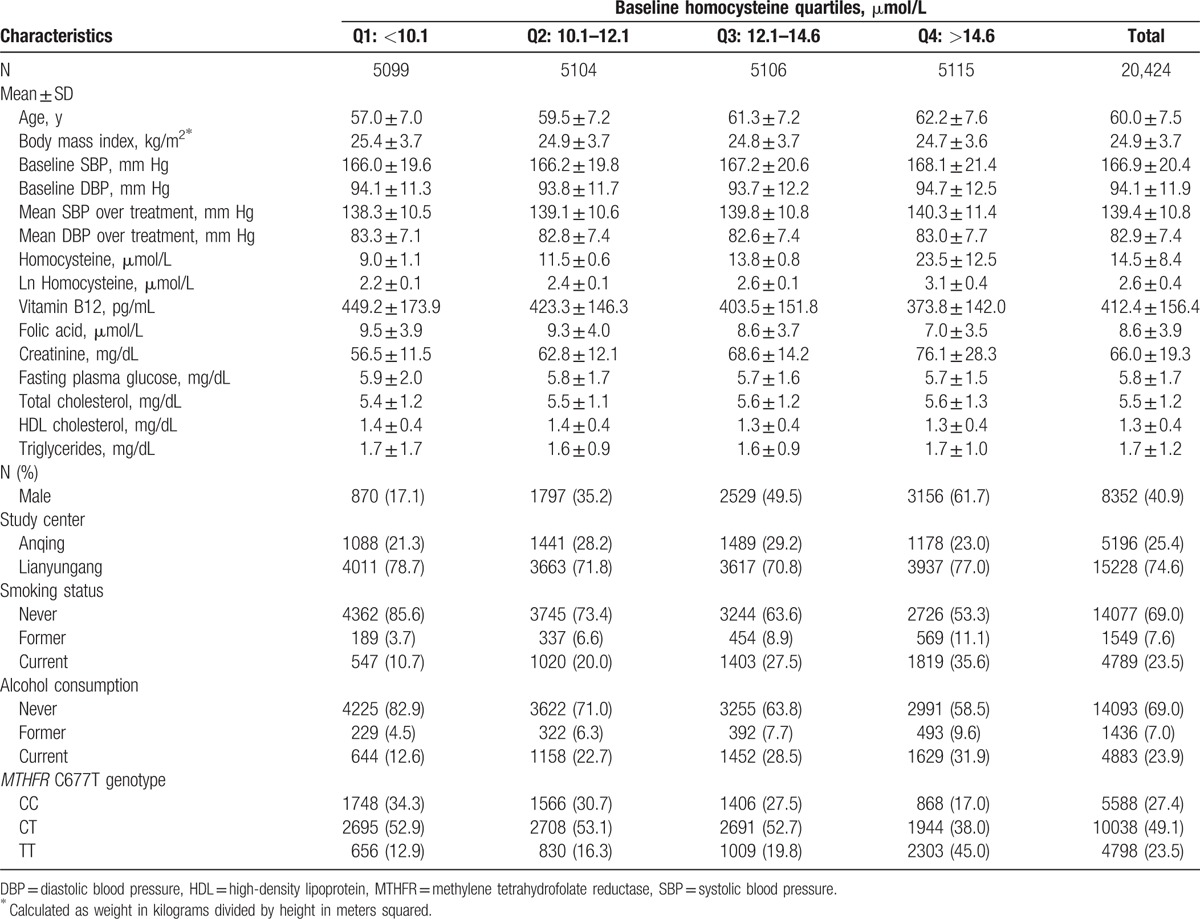
Characteristics of study population in total sample and stratified by total homocysteine quartiles.

### 3.2 Association between tHcy and all-cause mortality

**Figure 1:**
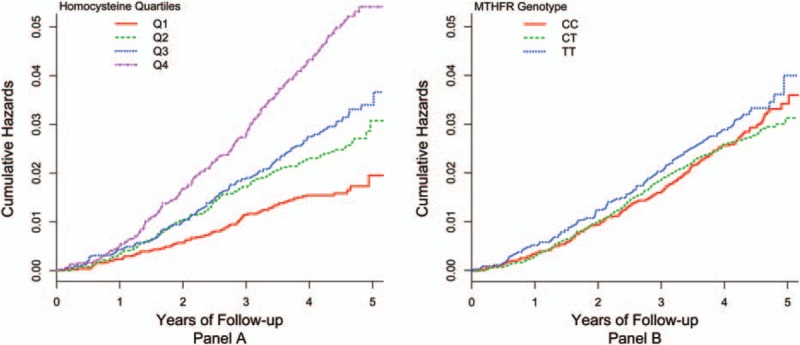
Kaplan–Meier curves for cumulative hazard of all-cause mortality. Panel A: stratified by quartiles of homocysteine; panel B: stratified by *MTHFR* genotype.

Among the 20,424 participants, there were 612 (3%) all-cause deaths during a mean treatment period of 4.5 years. Figure 1A presents the Kaplan–Meier curves for cumulative hazards of all-cause mortality, stratified by quartiles of tHcy. A dose–response relationship is evident between baseline tHcy and all-cause mortality; participants in the fourth quartile of tHcy had the highest risk, whereas those in the first quartile had the lowest risk. This relationship was further evaluated using the Cox proportional-hazards model, adjusting for age, sex, study center, baseline SBP and DBP, mean SBP and DBP, total cholesterol, triglycerides, HDL-C, fasting glucose, folate, B12, creatinine, BMI, smoking, alcohol use, and treatment group (Table 2). The HRs, using the lowest quartile as the reference, were 1.2, 1.2, and 1.5 in Q2, Q3, and Q4, respectively; however, significance was only attained in the fourth quartile (*P* < 0.01). A linear trend test using natural log-transformed tHcy resulted in an HR of 1.5 (95% CI 1.2–1.9), with a *P* < 0.001. The HRs were very similar after adjusting for the *MTHFR* genotype.

**Table 2:**
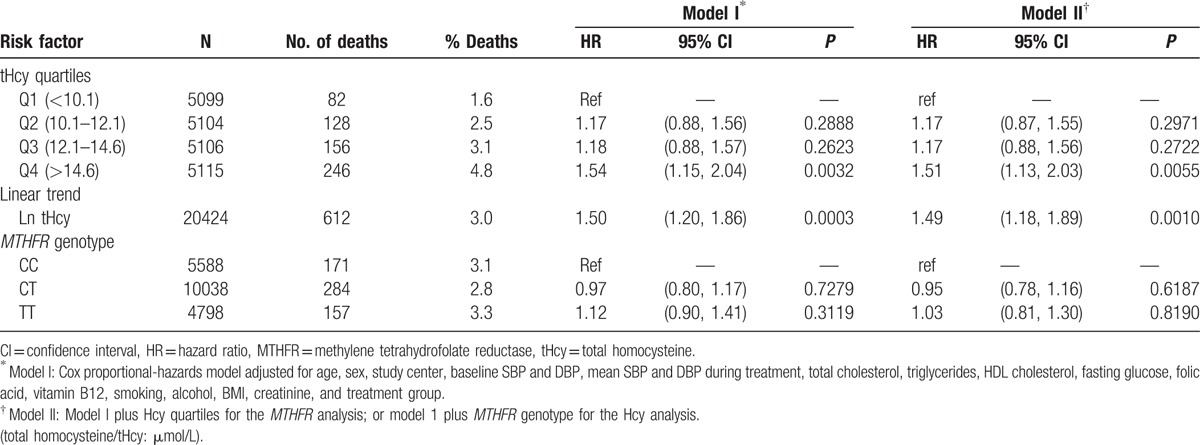
Association of baseline total homocysteine levels and *MTHFR* genotype with all-cause mortality.

### 3.3 Association between the MTHFR genotype and all-cause mortality

Figure 1B presents the Kaplan–Meier curves for cumulative hazards of all-cause mortality, stratified by *MTHFR* genotypes. Participants with the TT genotype had a slightly higher risk of death compared with the CC or CT genotypes, but the log-rank test was not significant. Consistently, there was no significant association between the *MTHFR* genotype, and mortality as evaluated by the Cox proportional-hazards model, adjusting for age, sex, study center, baseline SBP and DBP, mean SBP and DBP, total cholesterol, triglycerides, HDL-C, fasting glucose, folate, B12, creatinine, BMI, smoking, alcohol use, and treatment group (Table 2). The HRs were very similar after adjusting for tHcy.

### 3.4 Interaction of the MTHFR genotype and tHcy on mortality

**Figure 2:**
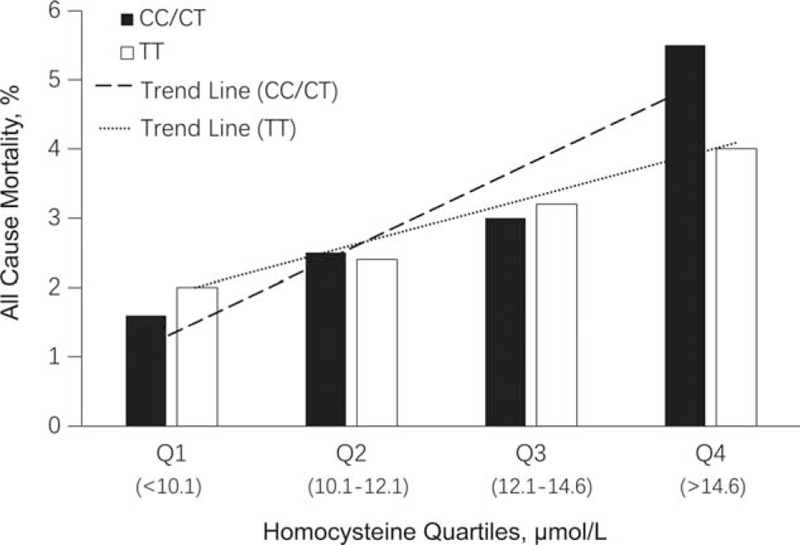
All-cause mortality by quartiles of homocysteine, Stratified by *MTHFR* genotype (CC/CT vs TT).

We investigated the potential modifying effect of the *MTHFR* genotype on the association between tHcy and mortality. Subjects with the CC and CT genotypes were grouped together and compared with TT genotypes. As shown in Fig. 2, although a dose–response trend is evident for both the CC/CT and TT groups, the linear slope was greater for the CC/CT group compared with the TT group. This differential association was further confirmed by Cox proportional-hazards modeling, adjusting for pertinent covariables (Table 3). A linear trend test using natural log-transformed Hcy resulted in an HR of 1.8 (95% CI 1.3–2.5, *P* < 0.001) for the CC/CT group, and 1.3 (95% CI 0.9–1.9, *P* = 0.23) for the TT group. The test for interaction between LnHcy and genotype (CC/CT vs TT) on all-cause mortality was significant (*P* = 0.04).

**Table 3:**
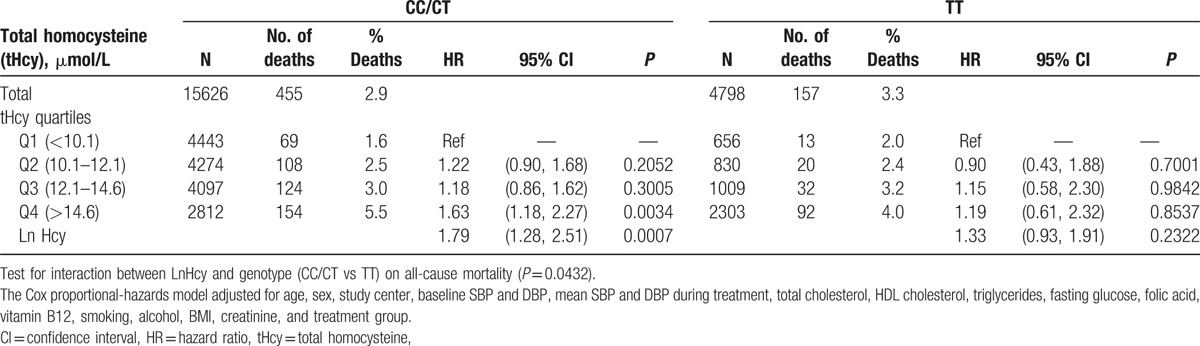
Interaction of total homocysteine levels and *MTHFR* C677T genotype on all-cause mortality.

### 3.5 Other effect modifiers on the tHcy–mortality relationship

We examined other potential effect modifiers on the association between tHcy and mortality (Supplemental Table 1). Whereas the general direction of the association was consistent across the strata of age, sex, baseline SBP, BMI, glucose, cholesterol, creatinine levels, and treatment group, the strength of the tHcy–mortality association varied across the strata. However, test for interaction was only significant for age (*P* < 0.001) and baseline creatinine (*P* < 0.01) strata.

## 4 Discussion

To our knowledge, this was the first and largest study to examine the longitudinal association between tHcy and a 5-year all-cause mortality in a Chinese hypertensive population, and to test the hypothesis that the tHcy0mortality association can be modified by the *MTHFR* C677T genotype.

This study, using data from the CSPPT, comprised the following unique features. First, the CSPPT included Chinese hypertensive participants without pre-existing stroke and/or MI. With a low vascular disease burden and a low frequency use of cardiac and vascular protective drugs, our results were less likely to be confounded by medications and vascular diseases. Second, the CSPPT participants had a much higher prevalence of elevated homocysteine (∼75%) and the *MTHFR* 677TT genotype (∼24%) than western populations, offering an exceptional opportunity to examine genetic modification with sufficient statistical power. Third, the CSPPT obtained data on individual *MTHFR* genotype for all participants and measured tHcy levels for 98.7% of participants. In contrast, many previous studies lacked individual *MTHFR* genotype data and/or only measured Hcy levels in a fraction of the participants, thereby, limiting their ability to test *MTHFR* genotype–Hcy interactions.^[21,22]^ This study has yielded important new knowledge as highlighted below.

### 4.1 Association of tHcy with all-cause mortality in hypertensive adults

Whereas a number of studies in western populations have consistently shown that elevated tHcy is a risk marker for all-cause mortality, the tHcy–mortality relationship in hypertensive patients has not been well-studied.^[8]^ Similarly, in China, a 12-year community-based prospective study of 2009 Chinese participants found that Hcy was associated with all-cause mortality.^[23]^ A recent study of 3799 Chinese patients with acute ischemic stroke found that elevated tHcy can predict 4-year mortality.^[24]^ Whereas, in general, these studies support tHcy as a predictor of mortality, no study has specifically examined the effect of tHcy on mortality in Chinese hypertensive patients.

Our study observed a dose–response relationship between baseline tHcy and all-cause mortality in Chinese hypertensive adults without pre-existing cardiovascular morbidities. This relationship persisted after adjusting for age, major cardiovascular, and metabolic covariates or confounders, including baseline creatinine level.^[14]^ This association also remained significant after further adjusting for the *MTHFR* genotype. Our data provide coherent evidence that elevated tHcy level is an independent risk factor of all-cause mortality in Chinese hypertensive patients without pre-existing cardiovascular and cerebral comorbidities.

### 4.2 Effect modification by the MTHFR C677T genotype

Hcy is a sulfur-containing amino acid produced by the demethylation of the essential amino acid methionine. The MTHFR C677T polymorphism is a major determinant of blood Hcy.^[25]^ However, questions remain to what degree the *MTHFR* genotype can modify the tHcy–mortality relationship.^[8]^

Our study found that the *MTHFR* genotype alone had little effect on mortality. Our finding is consistent with a US prospective study of 6000 adults,^[26]^ in which the *MTHFR* C677T TT genotype had no significant effect on all-cause mortality after adjustment for ethnic group and other CVD risk factors. Two meta-analyses also found no significant effect of the *MTHFR* genotype on vascular disease and longevity.^[27,28]^

Our study was the first to demonstrate a significant interaction between *MTHFR* C677T genotypes and tHcy on all-cause mortality: the strength of association between tHcy and mortality was significantly stronger in the CC/CT group than in the TT group.

The utility of the *MTHFR* 677TT genotype in Mendelian randomization was proposed in a previous study.^[16]^ However, our findings argue that the *MTHFR* C677T genotype should not be used as an independent marker in Mendelian randomization design, but rather should be incorporated to explore gene–environment interactions.

### 4.3 Biological mechanisms of tHcy in all-cause mortality

The underlying biological mechanisms for tHcy to impact all-cause mortality remain to be determined and may likely involve multiple pathways. First, mildly or moderately elevated tHcy may be a marker of suspected causal risk factors for death, including aging, B-vitamin deficiency, and chronic kidney disease. However, we found that the tHcy–mortality association remained after adjusting for age, folate, vitamin B12, and creatinine levels. Whereas the prevailing view of the pathogenesis of elevated tHcy is focused on cardiovascular events, the significant link between tHcy and all-cause mortality in hypertensive patients without pre-existing cardiovascular conditions strongly suggests a broader health effect. Indeed, hyperhomocysteinemia has been found to have a range of biological effects, including blunted nitric oxide bioactivity, enhanced generation of asymmetric dimethylarginine,^[29]^ and increased reactive oxygen species, all of which can lead to endothelial dysfunction.^[30]^ A better and more nuanced understanding of the molecular basis underlying hyperhomocysteinemia and pathogenic pathways may lead to new strategies to improve cardiovascular health, and also overall health and increase longevity.

### 4.4 Limitations

This study has limitations. Due to lack of data on specific causes of death, this study could not study cause-specific mortality. This study only assessed a 5-year mortality, and a second phase of the CSPPT with a longer follow-up is currently underway. This study focused on *MTHFR* C677T polymorphism; TT mutation is common in Chinese and is known to significantly reduce MTHFR enzyme activity. There is another mutation, A1298C, in *MTHFR* gene, which we did not measure. However, A1298C mutation is very low (3.9%) in Chinese and its impact on the enzyme activity is weak.^[31]^ Moreover, looking into other components in the homocysteine metabolic pathway, such as S-adenosylhomocysteine and adenosine, would provide further insight into homocysteine metabolism on mortality in the future.^[32]^

Despite its large sample size, this study was underpowered for evaluating higher-order interactions. A larger sample size is required to adequately assess other potential effect modifiers, including age, sex, baseline glucose levels, cholesterol, and creatinine or estimated glomerular filtration rate (eGFR), and concomitant use of other antidiabetic, antiplatelet, or antilipid medications. Additional analyses are needed to examine both baseline Hcy levels and Hcy lowering over the course of the trial on all-cause mortality. Although this study accounted for major covariables, the possibility of residual confounding cannot be excluded. Because the study population consisted of middle-aged or older Chinese hypertensive patients, caution is needed when generalizing the results to other populations.

### 4.5 Clinical and public health implications

All-cause mortality is one of the most basic and important indicators of a population's health, and is readily available from global and regional vital statistics. Although this study cannot provide a definitive answer on whether the observed tHcy–mortality association is causal, the study findings are consistent with available literature and offer new insight into the role of tHcy and its interaction with the *MTHFR* variant on all-cause mortality in hypertensive patients without pre-existing conditions. The study findings have important clinical and public health implications in light of high prevalence of hypertension and elevated tHcy in China and worldwide. In China alone, there are approximately 300 million hypertensive patients and about 1 billion in the world. If further confirmed that these study findings underscore the need to consider not only an individual's tHcy but also the *MTHFR* genotype to more precisely assess future mortality risk, representing 1 small step towards the goal of precision medicine.^[33]^

## 5 Conclusions

In this prospective analysis of Chinese adults with hypertension and without pre-existing cardiovascular and cerebral comorbidities, baseline tHcy predicted a 5-year all-cause mortality independent of major lifestyle, and cardiovascular and metabolic risk factors. Furthermore, the tHcy–mortality association was significantly greater in those with the *MTHFR* CC/CT genotype compared with those with the TT genotype. These data underscore the need to consider both tHcy levels and the *MTHFR* genotype as biomarkers for predicting future mortality risk in hypertensive patients.

## Supplementary Material

Supplemental Digital Content
