# Homocysteine and all-cause mortality in hypertensive adults without pre-existing cardiovascular conditions: Effect modification by MTHFR C677T polymorphism: Erratum

**DOI:** 10.1097/MD.0000000000007120

**Published:** 2017-06-02

In the article, “Homocysteine and all-cause mortality in hypertensive adults without pre-existing cardiovascular conditions: Effect modification by MTHFR C677T polymorphism”,^[1]^ which appeared in Volume 96, Issue 8 of *Medicine*, some of the authors’ degrees appear incorrectly. Benjamin Xu should not have an Bsc. Xiangyi Kong's degree should have appeared as an MD. Richard Xu should not have an Bsc. Lishun Liu should have an Bsc instead of an MS. Ziyi Zhou should have an Bsc instead of an MS.
