# Supplementary material for: Homocysteine and all-cause mortality in hypertensive adults without pre-existing cardiovascular conditions: Effect modification by MTHFR C677T polymorphism
Source: Medicine (Baltimore). 2017 Feb 24;96(8):e5862. doi: 10.1097/MD.0000000000005862 (PMC5569412; doi:10.1097/MD.0000000000005862)
Supplement: Supplemental Digital Content [file medi-96-e5862-s001.docx]

**Supplemental Table 1. Association between Total Homocysteine and All-Cause Mortality, Stratified by Other Factors**

| Factors* | N. Death/Sample Size, (%) | HR** | 95% CI | P-value | P for Interaction |
| --- | --- | --- | --- | --- | --- |
| Age |  |  |  |  | <0.0001 |
| ≤60 | 169/10326 (1.6) | 1.43 | (0.92, 2.24) | 0.1157 |  |
| >60 | 443/10098 (4.4) | 1.77 | (1.34, 2.22) | 0.0001 |  |
| Gender |  |  |  |  | 0.8058 |
| Male | 356/8352 (4.3) | 1.39 | (1.04, 1.87) | 0.0270 |  |
| Female | 256/12072 (2.1) | 1.70 | (1.09, 2.63) | 0.0187 |  |
| Baseline SBP, mmHg |  |  |  |  | 0.8193 |
| ≤163 | 261/9964 (2.6) | 1.33 | (0.91, 1.94) | 0.1418 |  |
| >163 | 351/10460 (3.4) | 1.59 | (1.17, 2.16) | 0.0033 |  |
| Body Mass Index |  |  |  |  | 0.2060 |
| ≤25 | 410/10836 (3.8) | 1.32 | (0.98, 1.78) | 0.0691 |  |
| >25 | 202/9580 (2.1) | 1.86 | (1.24, 2.80) | 0.0026 |  |
| Baseline Glucose, mg/dL |  |  |  |  | 0.3031 |
| ≤5.45 | 328/10198 (3.2) | 1.32 | (0.93, 1.86) | 0.1204 |  |
| >5.45 | 282/10118 (2.8) | 1.59 | (1.14, 2.22) | 0.0058 |  |
| Baseline Cholesterol, mg/dL |  |  |  |  | 0.7968 |
| ≤5.44 | 335/10216 (3.3) | 1.28 | (0.91, 1.80) | 0.1622 |  |
| >5.44 | 276/10102 (2.7) | 1.65 | (1.18, 2.32) | 0.0037 |  |
| Baseline Creatinine, mg/dL |  |  |  |  | 0.0050 |
| ≤63.7 | 215/10199 (2.1) | 1.45 | (0.91, 2.31) | 0.1147 |  |
| >63.7 | 396/10111 (3.9) | 1.81 | (1.38, 2.37) | <0.0001 |  |
| Treatment Group |  |  |  |  | 0.7777 |
| Enalapril | 314/10214 (3.1) | 1.58 | (1.15, 2.17) | 0.0050 |  |
| Enalapril-Folic Acid | 302/10210 (2.9) | 1.31 | (0.91, 1.89) | 0.1400 |  |
| Study Center |  |  |  |  | 0.7631 |
| Anqing | 216/5196 (4.2) | 0.96 | (0.56, 1.63) | 0.8765 |  |
| Lianyungang | 396/15228 (2.6) | 1.64 | (1.24, 2.15) | 0.0004 |  |

*All continuous variables were dichotomized at the median. **The effects were expressed as Hazard Ratios per ln unit of homocysteine by Cox proportional hazard models adjusting for age, sex, study center, baseline SBP and DBP, mean SBP and DBP during treatment, total cholesterol, HDL cholesterol, triglycerides, *MTHFR* C677T genotype, fasting glucose, folic acid, vitamin B12, smoking, alcohol, BMI, creatinine, and treatment group, except for the stratification variable.
